# Supplementary material for: Immunopeptidomics-based design of mRNA vaccine formulations against Listeria monocytogenes
Source: Nat Commun. 2022 Oct 14;13:6075. doi: 10.1038/s41467-022-33721-y (PMC9562072; doi:10.1038/s41467-022-33721-y)
Supplement: Supplementary file 1 — Supplementary Information [file 41467_2022_33721_MOESM1_ESM.pdf]

Supplementary Information

Immunopeptidomics-based design of mRNA vaccine formulations against *Listeria monocytogenes*

Rupert L. Mayer[1,2,3,$], Rein Verbeke[4,5], Caroline Asselman[1,2,6], Ilke Aernout[4,5], Adillah Gul[1,2], Denzel Eggermont[1,2], Katie Boucher[1,2,3], Fabien Thery[1,2], Teresa M. Maia[3], Hans Demol[1,2,3], Ralf Gabriels[1,2], Lennart Martens[1,2], Christophe Bécavin[7], Stefaan C. De Smedt[4,5], Bart Vandekerckhove[5,8], Ine Lentacker[4,5], Francis Impens[1,2,3]

[1] VIB-UGent Center for Medical Biotechnology, VIB, Ghent, Belgium.
[2] Department of Biomolecular Medicine, Ghent University, Ghent, Belgium.

[3] VIB Proteomics Core, VIB, Ghent, Belgium.

[4] Ghent Research Group on Nanomedicines, Ghent University, Ghent, Belgium.

[5] Cancer Research Institute Ghent (CRIG), Ghent, Belgium.

[6] Center for Medical Genetics, Ghent University Hospital, Ghent, Belgium

[7] Université Côte d'Azur, CNRS, IPMC, Sophia-Antipolis, France.

[8] Department of Diagnostic Sciences, Ghent University, 9000 Ghent, Belgium.

Correspondence: ine.lentacker@ugent.be and francis.impens@vib-ugent.be

[$]Present address: Research Institute of Molecular Pathology (IMP), Vienna BioCenter, Vienna, Austria.

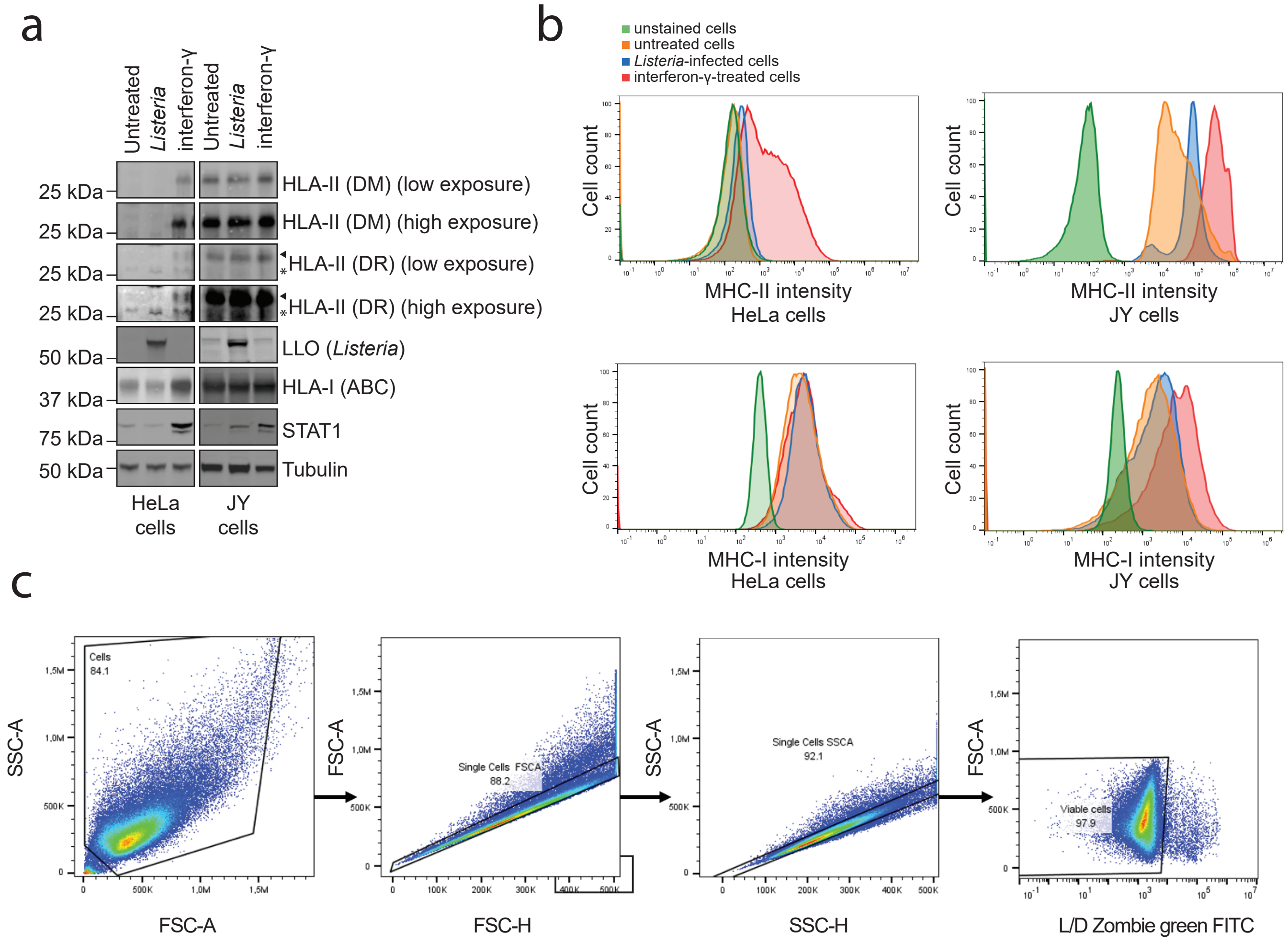

Supplementary Figure 1. HeLa cells do not express MHC-II during *Listeria* infection. (A-B) HeLa cells or JY cells (B cell line) were either infected with *Listeria monocytogenes* EGD (*Listeria*) for 24 h at MOI 25, or treated with 10 ng/mL interferon-γ for 48 h or left untreated. After infection, cells were either processed for western-blot (A) or flow cytometry (B) analysis. (A) MHC-I (anti-HLA-ABC) and MHC-II (anti-HLA-DM and -DR) were monitored by western blotting. Immunoblots against tubulin, listeriolysin O (LLO) and STAT1 serve as controls for loading, *Listeria* infection and interferon-γ treatment, respectively. (B) MHC-II (top panels) and MHC-I (bottom panels) were monitored by flow cytometry to determine their abundance on the cell surface in untreated, *Listeria* infected and interferon-γ-treated cells. Intensity signals for MHC-II and -I molecules are shown relative to unstained cells. Representative results of two independent experiments are shown. **(C)** Work flow describing the flow cytometry gating strategy used in (B). From left to right, exclusion of cell debris was performed by gating FSC-A versus SSC-A. This was followed by removal of cell aggregates by gating according to FSC-H/FSC-A parameters. Next, the removal of cell aggregates was performed by gating according to SSC-H/SSC-A parameters. Finally, dead cells were excluded from analysis by gating using Zombie green versus SSC-A parameter. Selected cells were analyzed to determine the abundance of MHC-II and –I molecule on cell surface. Source Data are provided at the end of this document.

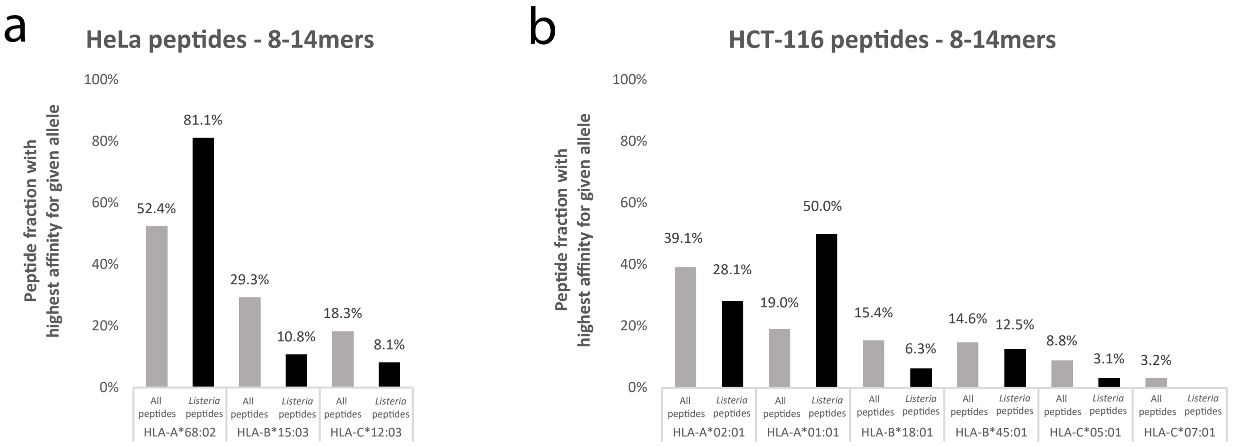

Supplementary Figure 2. *Listeria*-derived immunopeptides preferentially bind to HLA-A alleles. (A-B) For each 8-14mer immunopeptide, the HLA allele with the best % binding rank was determined using NetMHCpan 4.1 binding prediction (https://services.healthtech.dtu.dk/service.php?NetMHCpan-4.1). Interestingly, on both HeLa **(A)** and HCT-116 **(B)** cells the majority of immunopeptides showed the highest binding affinity for HLA-A alleles. This trend was even more pronounced for *Listeria*-derived immunopeptides of which ~80% showed strongest binding to HLA-A alleles in both HeLa and HCT-116 cells. Consequently, comparably fewer *Listeria* peptides showed high affinity for HLA-B and -C alleles. This could hint towards a favored presentation of bacterial peptides via HLA-A molecules, but further careful evaluation employing other pathogens and cell lines will be crucial to verify this hypothesis. Bar charts show the percentage of immunopeptides that are predicted to bind any of the three (HeLa cells) or six (HCT-116 cells) expressed HLA alleles (n=1 percentage for each allele, no statistical test applied). Source Data are provided as a Source Data file.

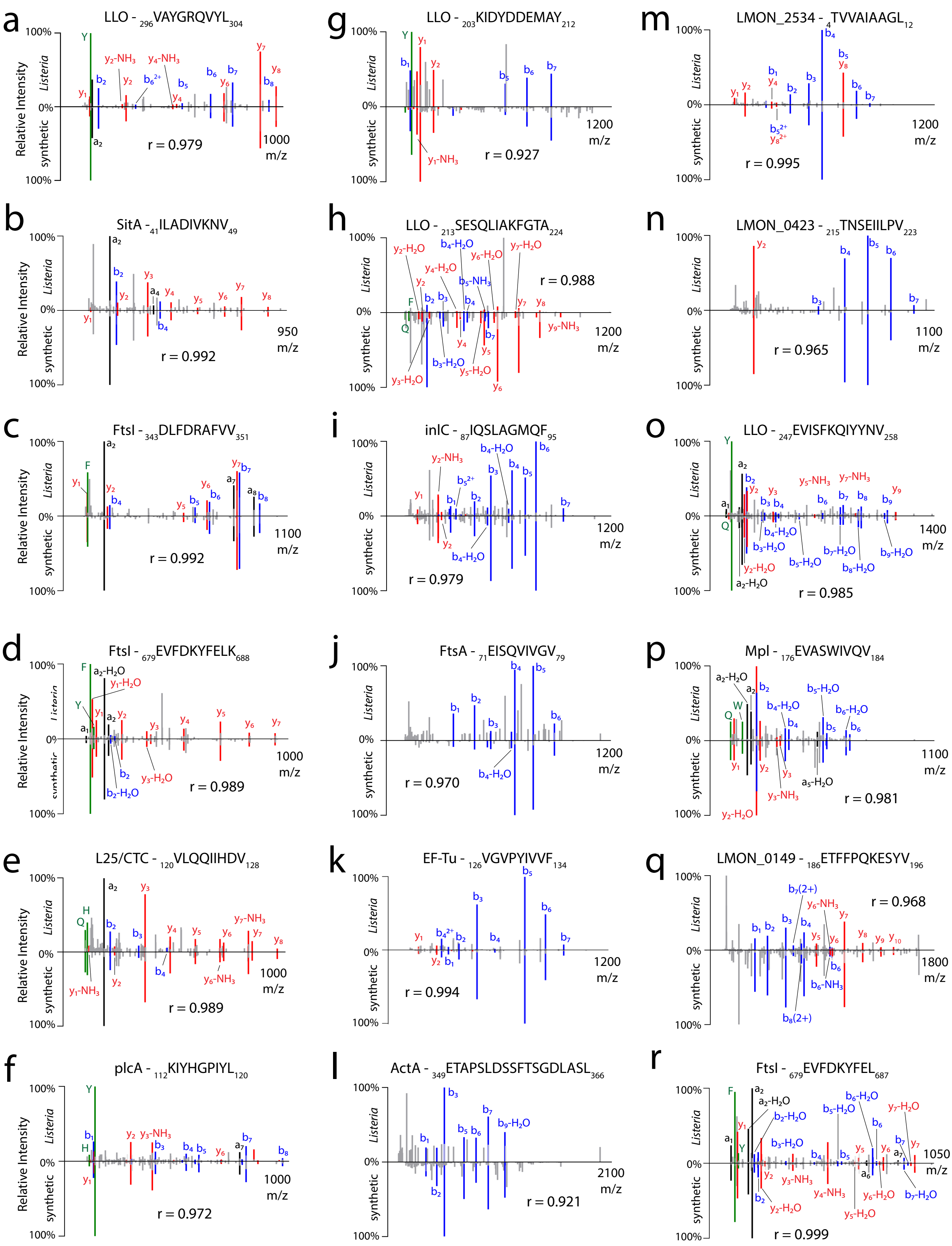
a
LLO - 296VAYGRQVYL304
r = 0.979
g
LLO - 203KIDYDDEMAY212
r = 0.927
m
LMON_2534 - 4TVVAIAAGL12
r = 0.995
b
SitA - 41ILADIVKNV49
r = 0.992
h
LLO - 213SESQLIAKFGTA224
r = 0.988
n
LMON_0423 - 215TNSEIILPV223
r = 0.965
c
FtsI - 343DLFDRAFVV351
r = 0.992
i
inlC - 87IQSLAGMQF95
r = 0.979
o
LLO - 247EVISFKQIYYNV258
r = 0.985
d
FtsI - 679EVFDKYFELK688
r = 0.989
j
FtsA - 71EISQVIVGV79
r = 0.970
p
Mpl - 176EVASWIVQV184
r = 0.981
e
L25/CTC - 120VLQQIIHDV128
r = 0.989
k
EF-Tu - 126VGVPYIVVF134
r = 0.994
q
LMON_0149 - 186ETFFPQKESYV196
r = 0.968
f
plcA - 112KIYHGPIYL120
r = 0.972
l
ActA - 349ETAPSLDSSFTSGDLASL366
r = 0.921
r
FtsI - 679EVFDKYFEL687
r = 0.999
Relative Intensity
Listeria
synthetic
100%
0%
m/z

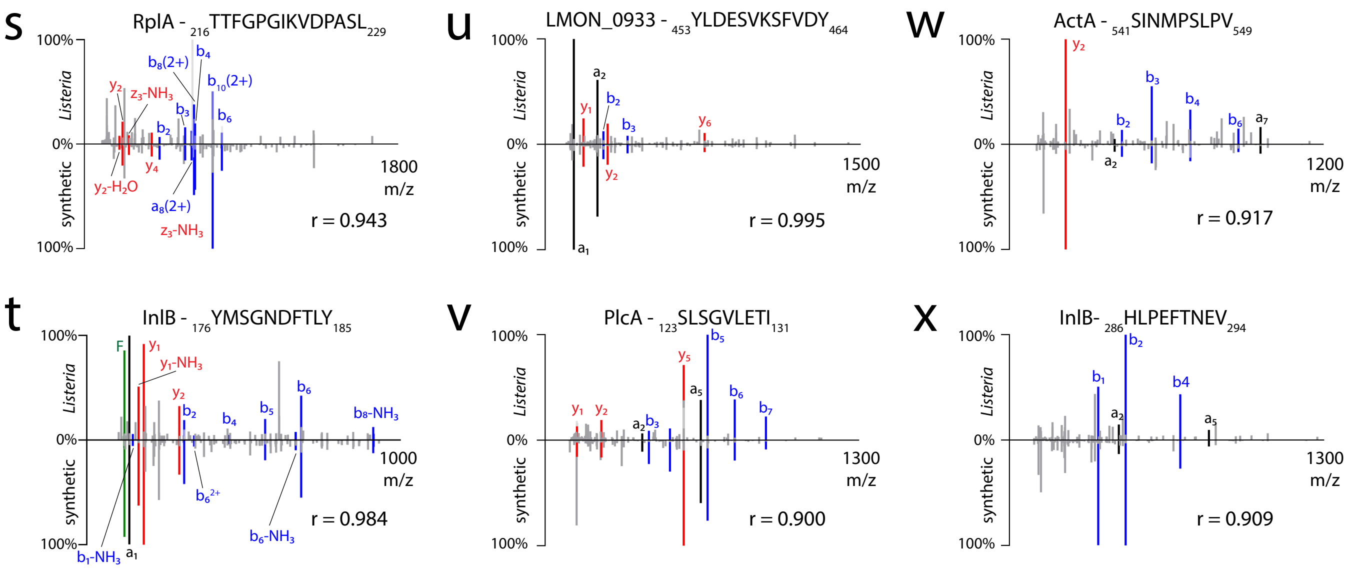

Supplementary Figure 3. Spectral comparison of *Listeria*-derived immunopeptides and their synthetic peptide counterpart. (A-X) In order to verify the identified *Listeria*-derived immunopeptide sequences, a selection of 24 peptides were synthesized and recorded under the same LC-MS/MS conditions as the infection-derived samples. For TMT-derived sequences, synthetic peptides were also TMT labeled prior to analysis. Fragmentation spectra per sequence were compared by plotting positive relative abundance values for *Listeria*-derived and negative relative abundances for synthetic peptides. Fragment ions assigned to the *Listeria*-derived immunopeptide sequences by PEAKS Studio that matched with fragment ions in the spectra of the corresponding synthetic peptide were highlighted. The high degree of overlap between synthetic and *Listeria*-derived fragmentation spectra corroborates sequence identity, further indicated by high Pearson correlations ($r \geq 0.90$) calculated for all peptides and indicated for all spectral comparisons (see Methods). Source Data are provided as a Source Data file.

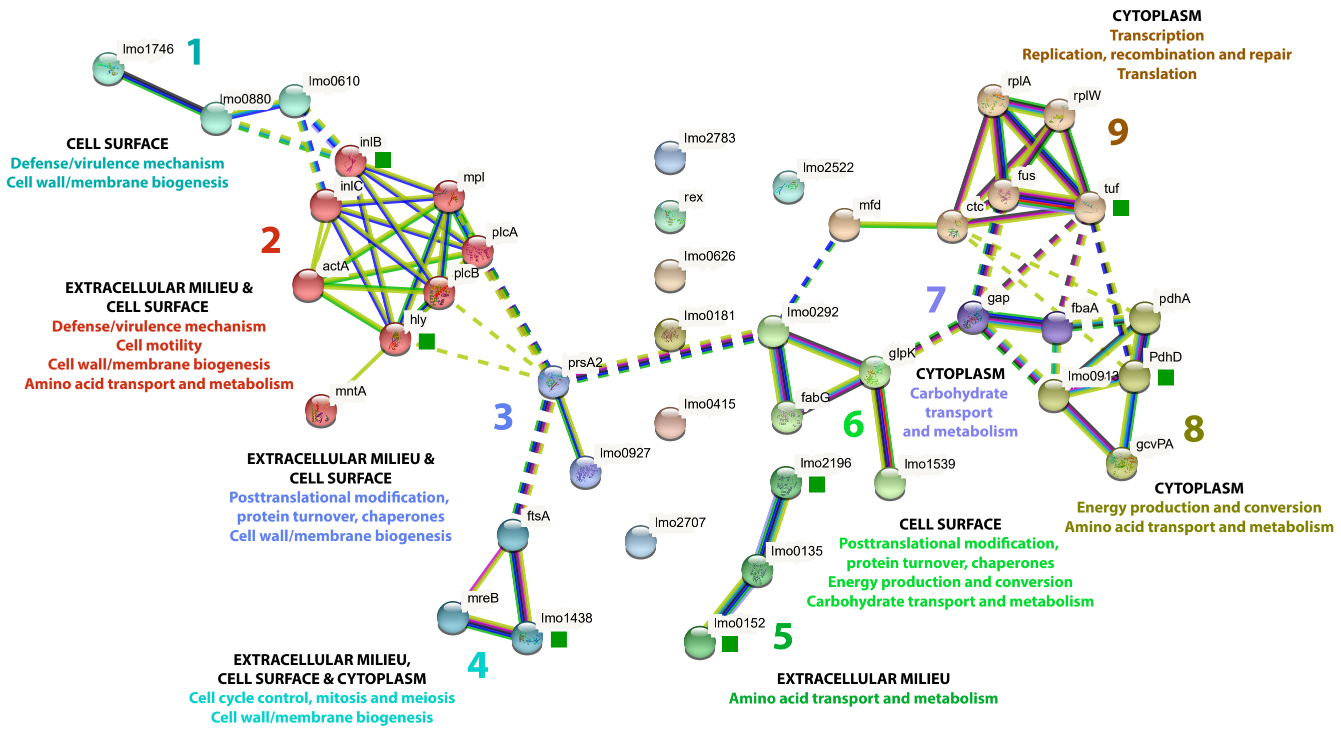

Supplementary Figure 4. STRING functional protein association. All 42 antigens of origin for the identified high confidence *Listeria* immunopeptides were subjected to the STRING database using their respective Lm EGDe homologs as input (due to Lm EGD not being available on STRING). Settings were kept at default (full STRING network, evidence based network edges, all interaction source to active, medium confidence (0.400) as minimum required interaction score, no interactors shown), and MCL clustering (inflation parameter 3.5) was selected as clustering method resulting in the annotation of nine clusters. Connections between clusters are visualized with dotted lines. Proteins chosen as vaccine candidates are depicted with green squares. Cluster 1 includes three proteins localized at the cell surface associated with defense/virulence mechanism and cell wall/membrane biogenesis. Connected to cluster 1, cluster 2 consists of eight proteins of which most are included in the pVGC with plcA, LLO, mpl, actA and plcB, also including inlB, inlC and mntA. Localized in the extracellular milieu or on the cell surface, these proteins are involved in defense/virulence mechanism and cell wall/membrane biogenesis, but also associated with cell motility and amino acid transport and metabolism. The two proteins of cluster 3 show the same subcellular localization and are also involved in cell wall/membrane biogenesis, but are furthermore associated with the COG term posttranslational modification, protein turnover, chaperones. Proteins from cluster 4 localize to the extracellular milieu, cell surface and cytoplasm and are involved in cell cycle control, mitosis and meiosis as well as cell wall/membrane biogenesis. Cluster 5, which is not connected to any other cluster, includes three OppA proteins involved in amino acid transport and metabolism localizing to the extracellular milieu. Cluster 6 interconnecting cluster 3, 7 and 9, consists of four proteins localized at the cell surface involved in posttranslational modification, protein turnover, chaperones as well as energy production and conversion and also carbohydrate transport and metabolism. Both proteins of cluster 7, gap and fbaA, participate in carbohydrate transport and metabolism and are localized in the cytoplasm (like all proteins for cluster 8 and 9 also). Cluster 8 encompasses four proteins that contribute to energy production and conversion as well as amino acid transport and metabolism. The six protein members of cluster 9 finally are involved in transcription as well as translation and also replication, recombination and repair. Source Data are provided in Supplementary Data 2.

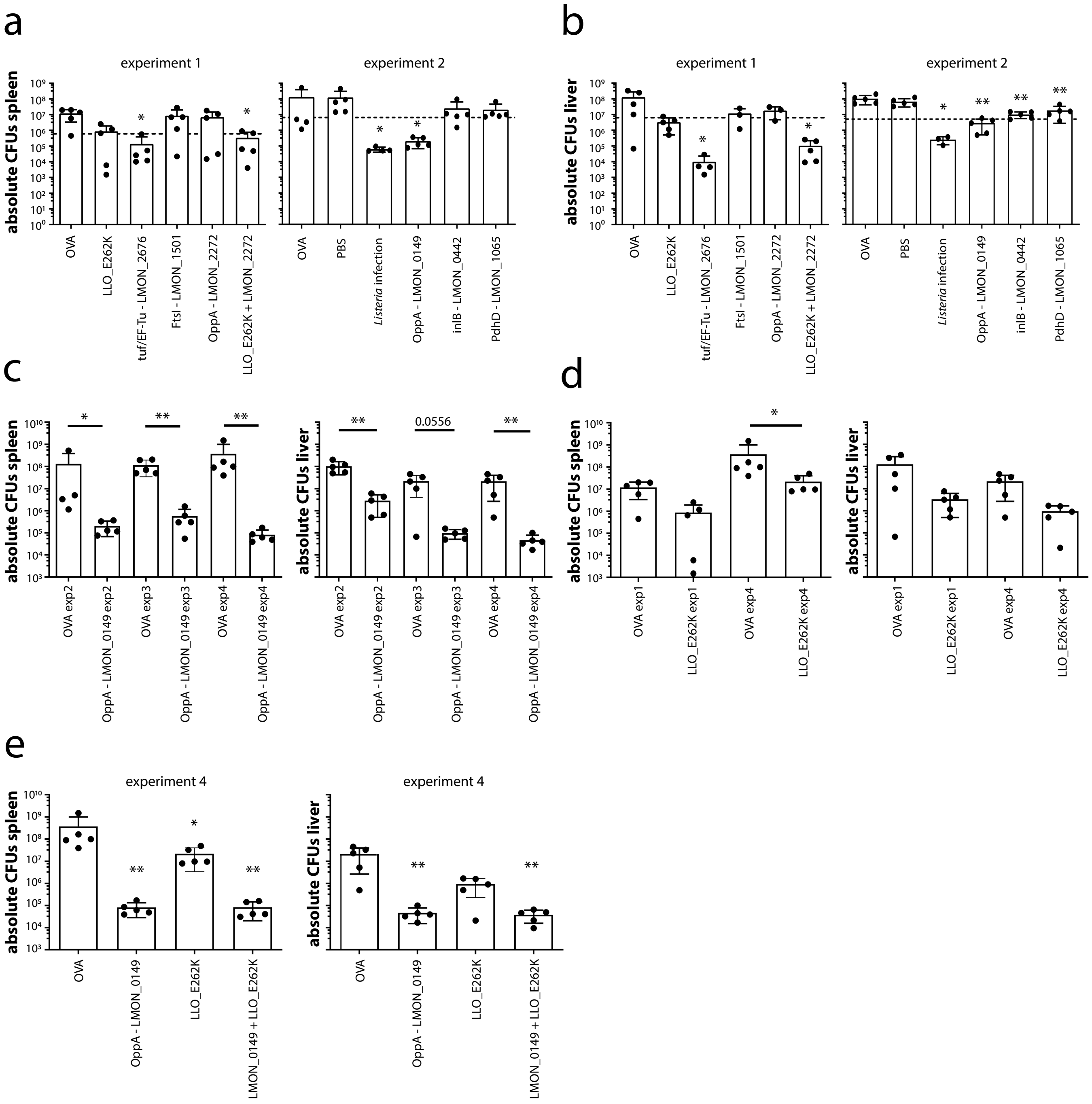

Supplementary Figure 5. Highly presented antigens provide protection as mRNA vaccine candidates. Next to the vaccination challenge experiments 1 and 2 shown in Figure 4, two additional independent experiments were performed using identical vaccine formulations for LMON_0149 and LLO_E262K (experiments 3 and 4). Experiment 4 further contained a vaccine formulation combining LMON_0149 and LLO_E262K. Number of mice/group was 5 in all 4 experiments. In experiment 1 and 2, no CFUs were detectable from a 1-2 biological replicates of a few conditions and were therefore excluded as indicated in the legend of Figure 4B. **(A-B)** Alternative visualization of the bar charts shown in Figure 4B, plotting non-normalized, absolute CFU values in log scale for experiments 1 and 2. **(C-D)** Bar charts depicting the non-normalized, absolute CFU counts measured for (C) LMON_0149 in experiments 2, 3 and 4 and for (D) LLO_E262K in experiments 1 and 4. LMON_0149 vaccination showed consistently high levels of protection in spleen (~3 log CFU reduction) and liver (~1.5 to 2.5 log CFU reduction). In contrast, protection by LLO_E262K was not significant in most cases with lower CFU reductions of ~1.5 logs in spleen and liver. **(E)** Combining LMON_0149 and LLO_E262K in a single vaccine formulation did not yield additional or synergistic protective effects, confirming the rather low protection of LLO_E262K in our vaccination platform. Source data are provided as a Source Data file.

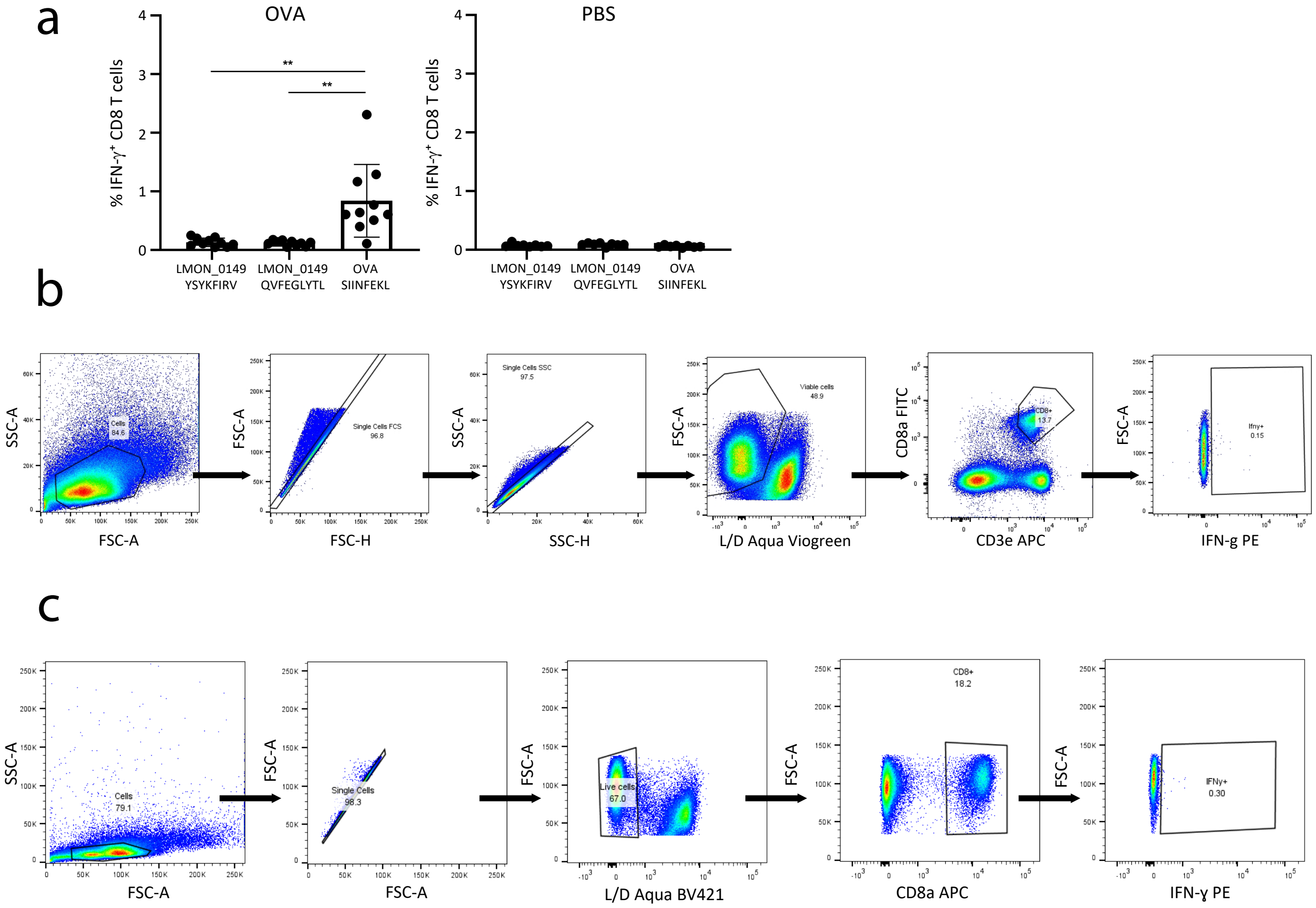

Supplementary Figure 6. Control T cell response measurements and flow cytometry gating strategy. (A-B) In an additional control experiment to the data shown in Figure 5, C57BL/6J mice were (A) vaccinated with mRNA galsomes encoding OVA or (B) injected with PBS (10 mice/group). After 7 days, splenocytes were isolated and pulsed with the OVA epitope SIINFEKL or the two LMON_0149 epitopes YSYKFIRV and QVFEGLYTL. No noticeable levels of IFN-γ$^+$ CD8 T cells could be detected upon pulsing with the LMON_0149 epitopes (average % IFN-γ$^+$ CD8 T cells < 0.14), while pulsing with the OVA-specific SIINFEKL peptide triggered statistically significant levels of IFN-γ$^+$ CD8 T cells in OVA-vaccinated mice (Shapiro-Wilk test rejected data normality, Wilcoxon matched-pairs signed rank test applied, data are presented as mean values +/- SD, n=10 individual animals for OVA and n=8 for PBS since splenocytes from 2 animals with <35% cell viability were excluded). Asterisks indicate p values with $^{**}p < 0.01$. Source data are provided as a Source Data file. These results further support the specificity of the responses measured for the LMON_0149 epitopes shown in Figure 5. **(B)** Gating strategy employed for the data shown in (A). Panels from left to right show i) exclusion of debris from cells by gating FSC-A versus SSC-A, ii) removal of cell aggregates by gating according to FSC-H/FSC-A parameters and iii) by gating according to SSC-H/SSC-A parameters, iv) exclusion of dead cells by gating only for live/dead aqua-low cells, v) gate used to identify CD3e CD8a double positive cells, vi) antigen-reactive CD8a cells were defined as IFN-ɣ+ expressing cells after peptide stimulation, and their frequencies were determined among total CD8a cells. The figure shows representative flow data of OVA vaccinated C57BL/6 mouse after pulsing with the QVFEGLYTL synthetic peptide. **(C)** Gating strategy used for the data presented in Figure 5. Panels from left to right show i) exclusion of debris from cells by gating FSC-A versus SSC-A, (ii) removal of cell aggregates by gating according to FSC-H/FSC-A parameters, iii) exclusion of dead cells by gating only for live/dead aqua-low cells, iv) gate used to identify CD8a positive cells, v) antigen-reactive CD8a cells were defined as IFN-ɣ+ expressing cells after peptide stimulation, and their frequencies were determined among total CD8a cells. The figure shows representative flow data of LMON_0149 vaccinated C57BL/6 mouse after pulsing with the QVFEGLYTL synthetic peptide. Source data are provided as a Source Data file.

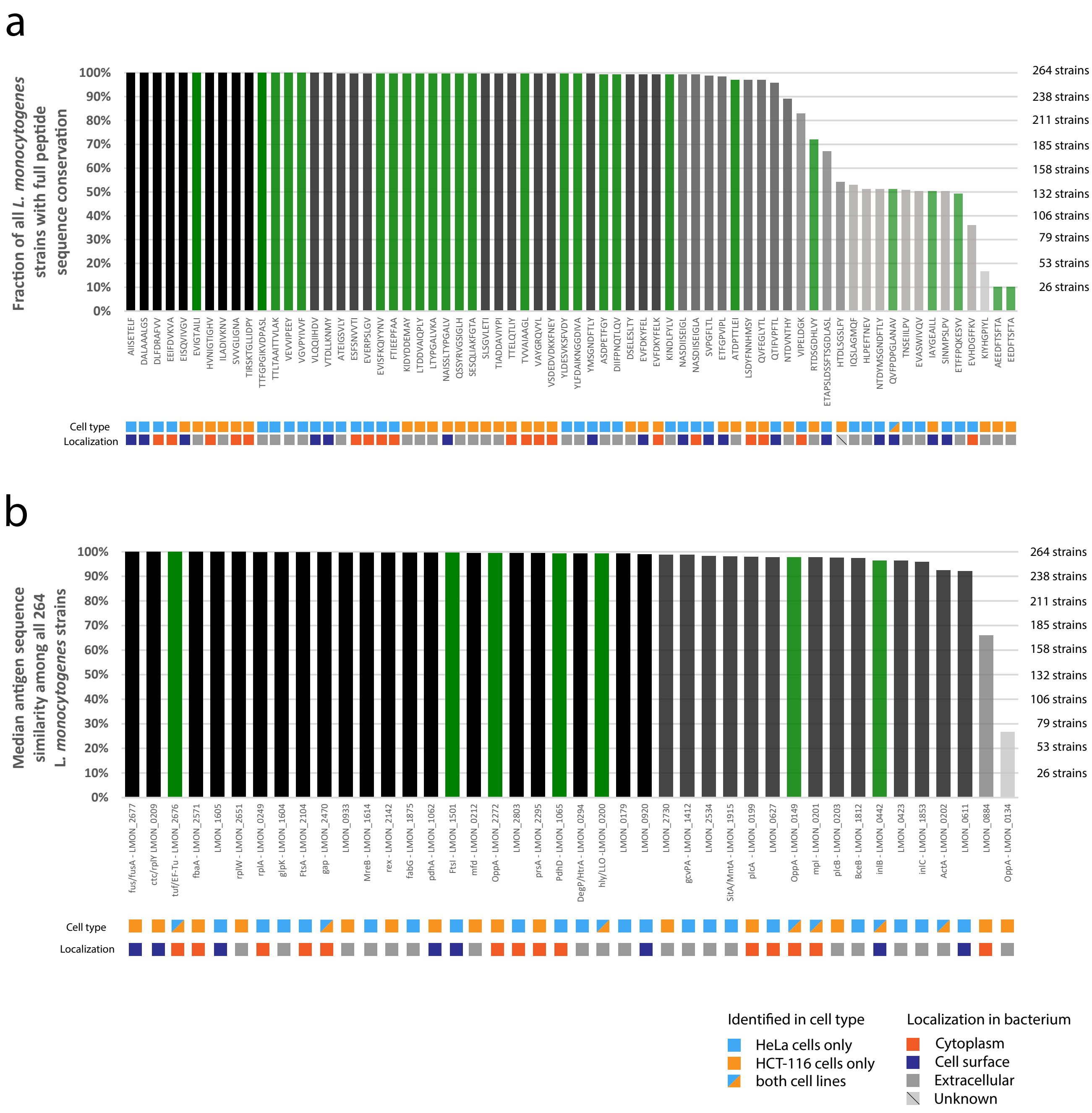

Supplementary Figure 7. Identified *Listeria* immunopeptides and antigens are well conserved. 264 fully sequenced *Listeria monocytogenes* strains were downloaded from NCBI RefSeq and used to create a BlastP database (Altschul et al. 1990) with all proteins annotated in these genomes. **(A)** We then ran BlastP (blast+ version 2.10) (Camacho et al. 2009) probing all 68 peptide sequences identified from our experiments with *Listeria monocytogenes* EGD versus all the proteins of each genome. BlastP parameters were deliberately set non-stringent (evalue 1000, max_target_seqs 1) to retrieve only perfect matches with mapping identity equal to 100%. For each immunopeptide we then plotted the percentage of strains in which the peptide sequence is fully conserved. Interestingly, the large majority (50 or 73%) of our identified peptides were fully conserved in more than 95% of the strains, while another 13 peptide sequences (19%) were fully conserved in at least 50% of the strains. Only five peptides were conserved in less than 50% of the investigated strains, indicating overall high conservation of the presented *Listeria* immunopeptides. **(B)** Denotes the antigen sequence similarity among all 264 *Listeria monocytogenes* strains, indicating greater than 90% sequence similarity for all but two antigens. For each antigen, the median percentage identity between the query sequence and the blast results is plotted. Antigens used in the mouse vaccination assays are depicted in green and show excellent sequence conservation of >95% in all strains. We therefore conclude that the chosen vaccine candidates will also hold substantial relevance not only for *Listeria monocytogenes* EGD, but also for more clinically relevant *Listeria* strains. Source data are provided as a Source Data file.

Supplementary Table 1 - Vaccination plasmid sequence information. All plasmid sequences used for the vaccine formulation production are listed. In short, protein sequences of the selected seven *Listeria* genes were cloned into a pGEM4z-plasmid vector (Promega) containing a T7 promoter, 5′ and 3′ UTR of human β globulin, and a poly(A) tail by Genscript. **Column A** depicts the plasmid name, **column B** the BUG number in our collection, **column C** the antigen gene name(s) and **column D** shows the plasmid sequence including the HindII/EcoRI restriction sites. **Column E** depicts the mRNA sequence produced from the T7 transcription start site of the pGEM-4z plasmid. For this the Nucleic Acid Converter (https://skaminsky115.github.io/nac/DNA-mRNA-Protein_Converter.html) was utilized using the T7 MegaScript kit (not including the Anti-Reverse Cap Analog (ARCA, Trilink BioTechnologies)). **Column F** illustrates the original amino acid sequence of the chosen antigens in Listeria monocytogenes EGD.

(A) Plasmid name	(B) Collection No.	(C) Antigen gene name(s)	(D) Plasmid insert sequence (including HindIII/EcoRI restriction sites on flanking ends, respectively)	(E) mRNA sequence produced from T7 transcription start site (https://skaminsky115.github.io/nac/DNA-mRNA-Protein_Converter.html) using the T7 MegaScript kit (not including the Anti-Reverse Cap Analog (ARCA, Trilink BioTechnologies))	(F) Antigen amino acid sequence
**pGEM-4z_OppA - LMON_0149**	**BUG101**	OppA<br>LMON_ 0149	[illegible]	[illegible]	MHFLKKTLPIFAILTVLLLTACGNDNEKSTTKTSPDKIKFLETSELLTLNTTAEEDFTSFTAQNQVFEGLYTLDQKDNFVPGVADGMPEISTDQTKYTIKLKKNAKWSDGSQVTADDFVYAWRRAVDPKTAPGYSALFKDSIKNATEINEGKLPVTDLGVVATDPTTLEITLKKPVPYFISLLSFETFFPQKESYVKKQGDKYGTDSAHTLYNGPFVMKDWGGNITNKWTYAKNDQYWDKDNVKVNEIDVQVAKDINAGVNLYNTNEADRVPLSGDFAKQYKDKKDFQTEKDALISYLRMNQKRDGKATPLANNSLRHALNLAVDKKQLTDRILGDGSFPANGLLPKDFVQNPTTGADFRTDSGDHLVYNKEEALKYWKQAQKELGTDKVTIELLGDDQETTKTIFAYLKAQFEDNLPGVTIKVKNMPSKSATQLTSDGNYDLSLAAWMPDFKDPWTYSSLFLSDYFNNHMSYNSPAYDKLVKSTDTTLATKPEERWNAFVASEKVLLDDDAAILPLYQHQTAVLQRTDITGVQKHAFGSPYSYKFIRVEK*
**pGEM-4z LLO_E26K2 - LMON_0200**	**BUG102**	LLO<br>hly<br>LMON_0200	[illegible]	[illegible]	MKKIMLVFITLILVSLPIAQQTEAKDASAFNKENSISSMAPPASPPASPKTPIEKKHADEIDKYIQGLDYNKNNVLVYHGDAVTNVPPRKGYKDGNEYIVVEKKKKSINQNNADIQVVNAISSLTYPGALVKANSELVENQPDVLPVKRDSLTLSIDLPGMTNQDNKIVVKNATKSNVNNAVNTLVERWNEKYAQAYPNVSAKIDYDDEMAYSESQLIAKFGTAFKAVNNSLNVNFGAISEGKMQEEVISFKQIYYNVNVNKPTRPSRFFGKAVTKEQLQALGVNAENPPAYISSVAYGRQVYLKLSTNSHSTKVKAAFDAAVSGKSVSGDVELTNIIKNSSFKAVIYGGSAKDEVQIIDGNLGDLRDILKKGATFNRETPGVPIAYTTNFLKDNELAVIKNNSEYIETTSKAYTDGKINIDHSGGYVAQFNISWDEVNYDPEGNEIVQHKNWSENNKSKLAHFTSSIYLPGNARNINVYAKECTGLAWEWWRTVIDDRNLPLVKNRNISIWGTTLYPKYSNKVDNPIE*
**pGEM-4z_EF-Tu - LMON_2676**	**BUG100**	EF-Tu<br>tuf<br>LMON_2676	[illegible]	[illegible]	MAKEKFDRSKPHVNIGTIGHVDHGKTTLTAAITTVLAKKGYADAQAYDQIDGAPEERERGITISTAHVEYQTDSRHYAHVDCPGHADYVKNMITGAAQMDGAILVVSAADGPMPQTREHILLSRQVGVPYIVVFMNKCDMVDDEELLELVEMEIRDLLTEYEFPGDDIPVIKGSALKALQGEADWEAKIDELMEAVDSYIPTPERDTDKPFMMPVEDVFSITGRGTVATGRVERGQVKVGDEVEVIGIEEESKKVVVTGVEMFRKLLDYAEAGDNIGALLRGVAREDIQRGQVLAKPGSITPHTNFKAETYVLTKEEGGRHTPFFNNYRPQFYFRTTDVTGIVTLPEGTEMVMPGDNIELAVELIAPIAIEDGTKFSIREGGRTVGAGVVSNISK*
**pGEM-4z_inlB - LMON_0442**	**BUG105**	inlB<br>LMON_0442	[illegible]	[illegible]	MKEKHNPRRKYCLISGLAIIFSLWIIIGNGAKVQAETITVSTPIKQIFPDDAFAETIKDNLKKKSVTDAVTQNELNSIDQIIANNSDIKSVQGIQYLPNVTKLFLNGNKLTDIKPLTNLKNLGWLFLDENKIKDLSSLKDLKKLKSLSLEHNGISDINGLVHLPQLESLYLGNNKITDITVLSRLTKLDTLSLEDNQISDIVPLAGLTKLQNLYLSKNHISDLRALAGLKNLDVLELFSQECLNKPINHQSNLVVPNTVKNTDGSLVTPEIISDDGDYEKPNVKWHLPEFTNEVSFIFYQPVTIGKAKARFHGRVTQPLKEVYTVSYDVDGTVIKTKVEAGTRITAPKPPTKQGYVFKGWYTEKNGGHEWNFNTDYMSGNDFTLYAVFKAETTEKTVNLTRYVKYIRGNAGIYKLPREDNSLKQGTLASHRCKALTVDREARNGGKLWYRLKNIGWTKAENLSLDRYDKMEYDKGVTAYARVRNASGNSVWTKPYNTAGAKHVNKLSVYQGKNMRILREAKTPITTWYQFSIGGKVIGWVDTRALNTFYKQSMEKPTRLTRYVSANKAGESYYKVPVADNPVKRGTLAKYKNQKLIVDCQATIEGQLWYRIRTSSTFIGWTKAANLRAQK*

**pGEM-4z_FtsI - LMON_1501**	**BUG099**	LMON_1501	aagcttAGACAGACATTTGCTTCTGACACAACTGTGTTCACTAGCAACCTCAAACAGAGGATCCCACC ATGAAAT[illegible] ATCCCCCTGCGGCTGAACATACTGTTCTTCATCATCTTTATCCTCTTTTCCGTACTGATACTCCGACTTGGCATCG[illegible] AATGTTACTGTAAGCAAAAATGTTCCACGCGGATCCATTTACGATCGAAACTACAATTTGCTGGTGGGCAATTC[illegible] AGACATTGCATGTCGCTCAAACCCTGGAAAAGCTGATTACTGTTGAACCTGAAAAATTGACCGATCGGGACTT[illegible] GCTAAGGAACAAGCACTTGACAGTTCCAAAGCATACAAAATACAAGTGGACAAGGTTACAAAAGAAGATATT[illegible] ACTACAGGATATGCAATGACTGAATCCGTGGTTAAGAACAAGGACGTTACAGATGAGGAAATTGCTCGGGTG[illegible] CGCTATTATACTTATGACGAAACTCTCAGAAGCATATTGGGGTCCGTATCCACTGCCAAAGAGGGCTTGCCAAA[illegible] TTGGGAAGTCCTACCTTGAAGCTCAGTACGAAAGCGTACTTGCAGGCAGTAAATCACAGTCCGAATCAGTCCT[illegible] AGGTAAGGATCTGGTTCTCAGCGTAGACGTCGAGTTCCAGAAAGCTGTGGAGGAAATACTTCAGAAGAACAT[illegible] TGTCGCAATGGACCCATATTCAGGTGAGGTGCTTGCCCTCGCTGGGCAGAAACTGAATGACAAAAGCGAGTT[illegible] GTGAAAGGTTCCACTATCCTGGGAGGGATAATGGATGGTGCAATCACAAACAAGACCGTGTTCACCGACCAG[illegible] GGAGCCGGTAATAGACCTCTCGATCCAGTCGGTGCCCTTGAAATCTCCAGTAATTCCTACATGTACCAGGTCG[illegible] CACTCTCCACATTTGACGATATGAGATACTACTACAACCAGTTCGGATTGGGTGTCAAGACCGGTATTGACCTG[illegible] TCTTGACTTTGCCATTGGTCAATATGATAGTTACACCCCACTGCAAATGGCTCAGTACGTAAGCACCATCGCCA[illegible] GTACTAACGGGGATTCTGTCGGTACTCTGGCTACCGCTAACGAACCAAAAGTATTGAACAAGATCGGCGTTAG[illegible] GAGTACTGGGACCGCTAGAACCGTTTTTACCAGCTCTGACTATGATGTAGCAGGAAAGACCGGAACCGCTGAT[illegible]	[illegible]	[illegible]
**pGEM-4z_OppA - LMON_2272**	**BUG104**	OppA<br>LMON_2272	[illegible]	[illegible]	[illegible]
**pGEM-4z_PdhD - LMON_1065**	**BUG103**	PdhD<br>LMON_1065	[illegible]	[illegible]	[illegible]

Source Data

Supplementary Figure 1 (left panels)

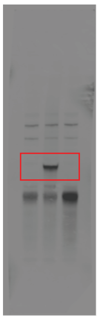

IB: anti-LLO

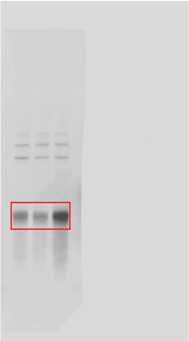

IB: anti-HLA-ABC

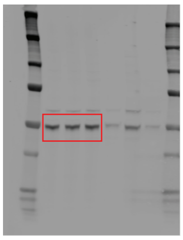

IB: anti-Tubulin-alpha

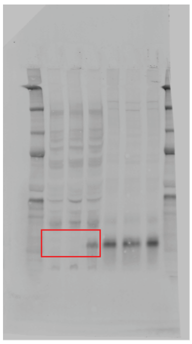

IB: anti-HLA-DM (DMB)
(low exposure)

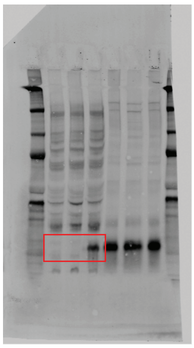

IB: anti- HLA-DM (DMB)
(high exposure)

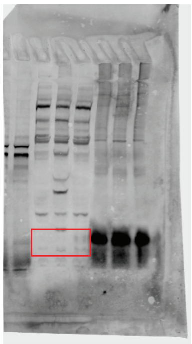

IB: anti-HLA-DR(DRB1)
(low exposure)

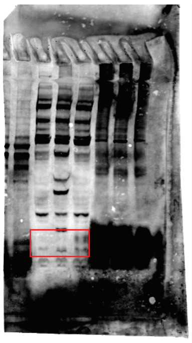

IB: anti-HLA-DR (DRB1)
(high exposure)

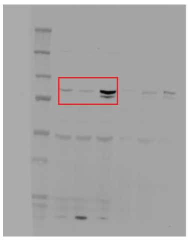

IB: anti-STAT1

Supplementary Figure 1 (right panels)

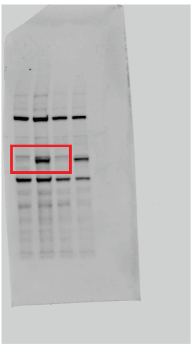

IB: anti-LLO

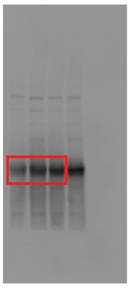

IB: anti-HLA-1-ABC

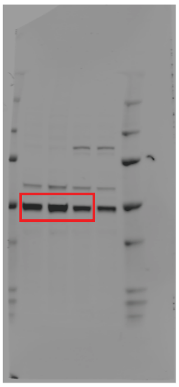

IB: anti-Tubulin-alpha

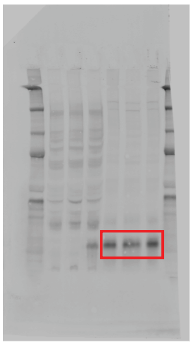

IB: anti-HLA-DM (DMB)
(low exposure)

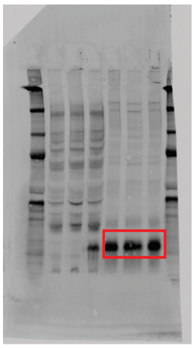

IB: anti- HLA-DM (DMB)
(high exposure)

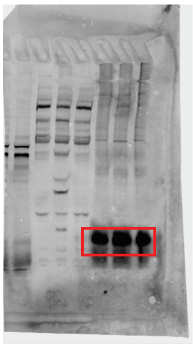

IB: anti-HLA-DR(DRB1)
(high exposure)

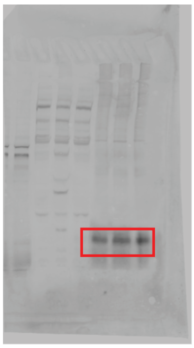

IB: anti-HLA-DR (DRB1)
(low exposure)

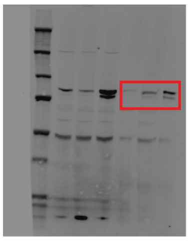

IB: anti-STAT1
