# Supplementary material for: Immunopeptidomics-based design of mRNA vaccine formulations against Listeria monocytogenes
Source: Nat Commun. 2022 Oct 14;13:6075. doi: 10.1038/s41467-022-33721-y (PMC9562072; doi:10.1038/s41467-022-33721-y)
Supplement: Supplementary file 3 — Description of Additional Supplementary Files [file 41467_2022_33721_MOESM3_ESM.pdf]

Description of Additional Supplementary Files

File Name: Supplementary Data 1
Description: **Quantified HeLa and HCT-116 peptide sequences.** Excel file with 8 tabs listing all quantified immunopeptides of the HeLa and HCT-116 infection experiments. Tabs list the results per cell line and quantification strategy or are concatenated per cell line. This sheet reports all immunopeptides quantified from both cell lines and quantification strategies. Listeria-derived peptides are denoted in green. Note that Listeria-derived peptides are not filtered for higher abundance in infected samples here.

File Name: Supplementary Data 2
Description: **High confidence Listeria epitopes.** All high confidence Listeria monocytogenes peptide epitopes retained after filtering out decoy sequences, potential contaminants, all potential host-derived sequences, as well as peptides not quantified in at least two infected samples and those with lower abundance in infected. Also Listeria sequences with leucine/isoleucine permutations found in the human database were discarded. After these filtering steps 68 bona fide Listeria peptide sequences were retained from 42 Listeria protein antigens. **Column A** displays the protein antigens name of origin of the identified epitopes in the Listeria monocytogenes EGD strain used for infection. **Column B and C** give the corresponding gene name and ORF name, respectively. For many less studied Listeria proteins these are identical. **Column D** shows the actual identified Listeria peptide sequence identified. **Column E and F** demonstrate gene names and protein names of homologs in the more deeply studied Listeria monocytogenes EGD-e. In **column G** the number of identified peptide sequences per protein antigen are described with 13 proteins giving rise to more than one peptide. **Column H** shows in which cellular system the peptide was identified, while **column I and J** indicate the start and endposition of the identified peptide wihtin the protein. Peptide length is given in **column K**. The PEAKS studio peptide identification score -10lgP indicates peptide identification confidence with higher values indicating greater confidence as shown in **column L**. Protein accession numbers are given in **column M**, whereas **column N and O** present the peptide fold change upon infection in label-free and TMT-labeling, respectively. **Column P** indicates by which method the peptide was identified: either LFQ, TMT or both. **Column Q** shows the Cluster of Orthologous Groups (COG) terms, representing a similar gene annotation strategy as GO terms, while being more informative in Listeria than GO terms. Protein accessions of the EGD-e strain homologs can be found in **column R**. In **column S** shows the predicted subcellular localization of the protein antigen according to Renier et al. 2012 PLoS One. Using NetMHCpan EL 4.1, for each peptide the highest affinity HLA allele per cell line was determined (**column T**). With the same method also the percentile rank in comparison to random peptide sequences was determined in **column U** yielding a high percentage of strong binders as anticipated corroborating the immunopeptide origin. **Column V and W** present the results of the IEDB recommended 2.22 MHC class II binding prediction model in order to assess if longer peptides could potentially constitute MHC class II ligands. **Column V** illustrates the MHC class II allele for which the peptide was found to have the highest MHC II binding affinity (=lowest percentile binding rank). For peptides identified in HeLa, binding predictions were performed against the MHC II alleles DQA1*01:02, DQB1*05:01 and DRB1*01:02 (alleles taken from http://celllines.tron-mainz.de/). MHC class II alleles for HCT-116 were derived from Boegel et al. 2014 Oncoimmunology with DQA1*05:02, DQB1*03:09, DQB1*02:02, DRB1*03:05, DRB1*11:02. However, no prediction model was available for DQA1*05:02 (the alpha chain of the heterodimer); therefore no predictions could be made for both the DQA1 and both DQB1 alleles as predictions can only be made for the heterodimer of DQA1 and DQB1 together. **Column W** depicts the peptide-length adjusted percentile binding rank in comparison to a random peptide list. Strong binders are characterized typically have percentile ranks <1%, weak binders 1-5% and non-binders >5% for MHC

class II molecules. According to these threshold, all Listeria-derived peptides are classified as MHC II non-binders, which further supports their identity as MHC class I ligands.

File Name: Supplementary Data 3
Description: **Mouse body weights during vaccination and Listeria challenge.** Mice were weighed daily before and up until three days post-vaccination. **Column A** indicates the experiment number as two vaccination experiments at two different times were carried out. **Column B** indicates the injected antigen(s), while **column C** shows the mouse ID. **Column D** depicts the initial weight of each mouse before any injection. **Column E** presents mice weights one day after prime vaccination in gram, while **column F** depicts mouse weights one day post-prime vaccination in % of pre-prime weight. **Column G** presents the day 1 post-prime average weight % of the pre-prime weight. **Column H** presents mice weights two days after prime vaccination in gram, while **column I** depicts mouse weights two days post-prime vaccination in % of starting weight. **Column J** presents the day 2 post-prime average weight % of the pre-prime weight. **Column K** presents mice weights three days after prime vaccination in gram, while **column L** depicts mouse weights three days post-prime vaccination in % of starting weight. **Column M** presents the day 3 post-prime average weight % of the pre-prime weight. **Columns N-AG** follow the same scheme only for the booster vaccination and the Listeria challenge.

File Name: Supplementary Data 4
Description: **Peptide binding prediction for lmon_0149 and C57BL/6J mouse MHC I molecules.** Peptide binding prediction for lmon_0149 was carried out for peptide lengths 8 and 9 and C57BL/6J mouse alleles H2-Db and H2-Kb using the IEDB analysis resource NetMHCpan (ver. 4.1) tool (http://tools.iedb.org/mhci/, Reynisson et al., 2020). Probability of natural peptide processing for lmon_0149 was carried out also for both 8 and 9mers and both H2-Db and H2-Kb alleles using MHC-NP (http://tools.iedb.org/mhcnp/, Giguère et al., 2013) Strong binders (percentile rank <0.5) for all lengths and alleles were retained and two peptides chosen for T cell stimulation experiments of vaccinated mice according to percentile rank, MHC-NP prediction and binding to both alleles. Column A idicates the alleles to which the peptide was predicted to bind, while column B indicates the number of proteins the prediction was made for. Start and end (column C and D) illustrate the position of the peptide within the protein. Peptide length and sequence are presented in columns E and F. Columns G denotes the peptide's identified binding core, which is always nine amino acids long and is used for sequence alignment and identification of binding anchors. Column H is a substring of the peptide that encompasses all residues between P1 and P-Omega of the MHC. Column I shows the peptide binding score with higher scores indicating higher affinity. The percentile rank in column J depicts the percentage of randomly sampled peptides scoring better than this specific peptide. Column K illustrates the MHC-NP results with higher numbers indicating higher probability of natural processing by the MHC pathway.
