# Supplementary material for: Immunopeptidomics-based design of mRNA vaccine formulations against Listeria monocytogenes
Source: Nat Commun. 2022 Oct 14;13:6075. doi: 10.1038/s41467-022-33721-y (PMC9562072; doi:10.1038/s41467-022-33721-y)
Supplement: Supplementary file 8 — Reporting Summary [file 41467_2022_33721_MOESM8_ESM.pdf]

Corresponding author(s): Francis Impens

Last updated by author(s): Sep 23, 2022

Reporting Summary

Nature Portfolio wishes to improve the reproducibility of the work that we publish. This form provides structure for consistency and transparency in reporting. For further information on Nature Portfolio policies, see our Editorial Policies and the Editorial Policy Checklist.

Statistics

For all statistical analyses, confirm that the following items are present in the figure legend, table legend, main text, or Methods section.

n/a	Confirmed	
☐	☒	The exact sample size (*n*) for each experimental group/condition, given as a discrete number and unit of measurement
☐	☒	A statement on whether measurements were taken from distinct samples or whether the same sample was measured repeatedly
☐	☒	The statistical test(s) used AND whether they are one- or two-sided<br>*Only common tests should be described solely by name; describe more complex techniques in the Methods section.*
☒	☐	A description of all covariates tested
☐	☒	A description of any assumptions or corrections, such as tests of normality and adjustment for multiple comparisons
☐	☒	A full description of the statistical parameters including central tendency (e.g. means) or other basic estimates (e.g. regression coefficient) AND variation (e.g. standard deviation) or associated estimates of uncertainty (e.g. confidence intervals)
☐	☒	For null hypothesis testing, the test statistic (e.g. *F*, *t*, *r*) with confidence intervals, effect sizes, degrees of freedom and *P* value noted<br>*Give P values as exact values whenever suitable.*
☒	☐	For Bayesian analysis, information on the choice of priors and Markov chain Monte Carlo settings
☒	☐	For hierarchical and complex designs, identification of the appropriate level for tests and full reporting of outcomes
☐	☒	Estimates of effect sizes (e.g. Cohen's *d*, Pearson's *r*), indicating how they were calculated

Our web collection on statistics for biologists contains articles on many of the points above.

Software and code

Policy information about availability of computer code

Data collection	Mass spectrometry data of immunopeptides was acquired on a Q Exactive HF or Fusion Lumos system (Thermo Scientific). Flow cytometry data of peptide pulsed splenocytes was acquired on a MACSQuant 16 flow cytometer (BD Biosciences).
Data analysis	Proteomics data analysis was performed with PEAKS Studio X+ (Bioinformatics Solutions Inc, version 10.5 build 20200219) and Perseus 1.6.2.1. All data were tested for statistical significance with GraphPad Prism 9.3. Flow cytometry data was analyzed with FlowJo 10.7.1. software (BD Biosciences). Human and Listeria databases were concatenated using dbtoolkit dbtoolkit 2.0 (version 4.2.5).<br><br>Python 3.7 was used to calculate spectral correlation between Listeria-derived and synthetic peptides including spectrum_utils version 0.3.5 and pyteomics version 4.5.2. The code including an example peptide can be found on GitHub (https://github.com/RalfG/2022-listeria-spectrum-similarity) and Zenodo (https://doi.org/10.5281/zenodo.5948475). A runnable version of the script can be found online at Binder (https://mybinder.org/v2/gh/RalfG/2022-listeria-spectrum-similarity/HEAD?labpath=2022-listeria-spectrum-similarity.ipynb).<br><br>The Vaxign-ML module of the Vaxign2 vaccine design platform was used for in silico evaluation of the selected vaccine candidates by means of reverse vaccinology.

For manuscripts utilizing custom algorithms or software that are central to the research but not yet described in published literature, software must be made available to editors and reviewers. We strongly encourage code deposition in a community repository (e.g. GitHub). See the Nature Portfolio guidelines for submitting code & software for further information.

Data

Policy information about availability of data

All manuscripts must include a data availability statement. This statement should provide the following information, where applicable:

- Accession codes, unique identifiers, or web links for publicly available datasets
- A description of any restrictions on data availability
- For clinical datasets or third party data, please ensure that the statement adheres to our policy

The mass spectrometry proteomics data have been deposited to the ProteomeXchange Consortium via the PRIDE partner repository with the dataset identifier PXD031451. The UniProt SwissProt and TrEMBL databases were accessed via https://www.uniprot.org/, while the IEDB database and associated analysis resource tools NetMHCpan v4.1 and MHC-NP were accessed via https://www.iedb.org/, http://tools.iedb.org/mhci/ and http://tools.iedb.org/mhcnp/, respectively. Data supporting the findings of this manuscript are available within the article, the Supplementary Data, the Supplementary Information and the Source Data files.

Field-specific reporting

Please select the one below that is the best fit for your research. If you are not sure, read the appropriate sections before making your selection.

☒ Life sciences ☐ Behavioural & social sciences ☐ Ecological, evolutionary & environmental sciences

For a reference copy of the document with all sections, see nature.com/documents/nr-reporting-summary-flat.pdf

Life sciences study design

All studies must disclose on these points even when the disclosure is negative.

Sample size	No statistical methods were used to predetermine sample size for experimentation. Given the minimal variation in clonal cell lines and lab mice, immunopeptidomics proteomics experiments included 4 replicates/condition and animal experiments contained at least 5 animals/group. For immunopeptidomics, the difference between non-infected and infected cell cultures was estimated to be considerable due to high levels of infection in the Listeria-challenged cultured compared to uninfected cultures. Hence, a set of four biological replicates was considered sufficient. Based on the higher level of biological variation in mice, a larger sample size of 5 animals for vaccination-challenge experiments and 10 animals for T cell response experiments was approximated to be sufficient, as evidenced by the achieved statistical significance in the end.
Data exclusions	No data points were excluded, except in the flow cytometry data analysis of peptide pulsed splenocytes. Here, data points with <35% cell viability were excluded as indicated in the figure legends and methods section.
Replication	Experiments contained at least 4 replicates to be sure that the data shown in the paper is reproducible. Immunopeptidomics experiments on HeLa and HCT-116 cells were carried out with 4 replicates/condition, while animal experiments contained 5 mice/group for the vaccination-challenge experiments and 10 mice/group for the vaccination experiments followed by peptide pulsing of isolated splenocytes.
Randomization	Cells and animals were randomly allocated to experimental groups.
Blinding	No blinding was applied in the study. For the immunopeptidomics samples no blinding was performed due to the expected large quantitative differences in Listeria-derived immunopeptides and an otherwise potential risk of carry-over when samples would be grouped for LC-MS/MS analysis in a mixed fashion.

Reporting for specific materials, systems and methods

We require information from authors about some types of materials, experimental systems and methods used in many studies. Here, indicate whether each material, system or method listed is relevant to your study. If you are not sure if a list item applies to your research, read the appropriate section before selecting a response.

Materials & experimental systems

n/a	Involved in the study	
☐	☒	Antibodies
☐	☒	Eukaryotic cell lines
☒	☐	Palaeontology and archaeology
☐	☒	Animals and other organisms
☒	☐	Human research participants
☒	☐	Clinical data
☒	☐	Dual use research of concern

Methods

n/a	Involved in the study	
☒	☐	ChIP-seq
☐	☒	Flow cytometry
☒	☐	MRI-based neuroimaging

Antibodies

Antibodies used	W6/32 antibody for pull down of MHC Class I:peptide complexes was purified in-house from hybridoma cell HB-95™ (ATCC) supernatant as recommended by the cell line provider. The antibodies used for flow cytometry of T cell responses were APC or FITC conjugated anti-mouse CD8a (1:100, Biolegend 53-6.7, #100712 and #100706, Lot B308246 and B312822), APC conjugated anti-mouse TCRbeta (1: 100, Thermo Fisher Scientific, H57-597, #17596182, Lot 1993624) and PE conjugated anti-mouse IFN-ɣ (1:50, Biolegend, XMG1.2, #505808, Lot B335691). The antibodies used to detect MHC-I and MHC-II by flow cytometry were PE conjugated anti-human HLA-ABC (1:100, Biolegend W6/32, #311405, Lot B327578) and APC conjugated anti-human HLA-DR/DP/DQ (1:100, Biolegend, Tü39, #361713, Lot B352671). The primary antibodies used to detect MHC-I and MHC-II by western blotting were anti-HLA-ABC (1:1000, Proteintech, #15240-1-AP), anti-HLA-DM (1:1000, Proteintech, #21704-1-AP), anti-HLA-DR (1:1000, Proteintech, #15862-1-AP), anti-α-tubulin (1:1000, Santa Cruz Biotechnology, #sc-5286), anti-Listeriolysin O (LLO) (1:1000, Abcam, #ab200538) and anti-STAT1 (1:1000, Santa Cruz Biotechnology, #sc-464). Secondary antibodies were anti-mouse (1:5000, Li-COR, #926-32210) and anti-rabbit (1:5000, Li-COR, # 926-32211).
Validation	The W6/32 antibody was validated by flow cytometry after staining JY cells (B-cell line). All commercial antibodies were used according to manufacturers' instructions without further validation by the authors.<br><br>APC or FITC conjugated anti-mouse CD8a and APC conjugated anti-mouse TCRbeta were validated by the manufacturers by flow cytometry after staining C57BL/6 mouse splenocytes. PE conjugated anti-mouse IFN-ɣ was similarly validated after staining BALB/c T-cells, while PE conjugated anti-human HLA-ABC and APC conjugated anti-human HLA-DR/DP/DQ were validated after staining human peripheral blood lymphocytes.<br><br>anti-HLA-ABC, anti-HLA-DM and anti-HLA-DR were validated by the manufacturer by immunohistochemistry of human tonsillitis tissue and by western blotting and/or immunoprecipitation from lysates of A549, HEK-293, HeLa, HepG2 (anti-HLA-ABC) or Raji or Daudi cells (anti-HLA-DM/DR). anti-Listeriolysin O (LLO) has been validated by the manufacturer for western blotting and the same holds true for anti-STAT1 that was validated by western blotting against whole cell lysates of HUV-EC-C, A-431 and K-562 cells.

Eukaryotic cell lines

Policy information about cell lines

Cell line source(s)	HeLa cells (ATCC® CCL-2™), HCT-116 cells (ATCC® CCL-247™), W6/32 (ATCC® HB-95™) and JY cells (ATCC® 77441™) were purchased from ATCC.
Authentication	HeLa and HCT-116 cells were authenticated by PCR single locus technology by Eurofins on March 20, 2019. JY and W6/32 cells were not authenticated.
Mycoplasma contamination	We consistently and regularly test for Mycoplasma using kits and PCR which is even more sensitive. All of our lines are negative.
Commonly misidentified lines (See ICLAC register)	We used only cell lines that were derived from ATCC. None of these cell lines are listed as commonly misidentified on ICLAC.

Animals and other organisms

Policy information about studies involving animals; ARRIVE guidelines recommended for reporting animal research

Laboratory animals	We used female C57BL/6J mice ordered from Charles River Laboratories, France, at 7 weeks of age for all experiments. The animals were housed in a temperature- (21°C) and humidity- (60%) controlled environment with 12h light/dark cycles; food and water were provided ad libitum.
Wild animals	The study did not involve wild animals.
Field-collected samples	The study did not involve samples collected from the field.
Ethics oversight	The authors confirm that all animal experiments were done under conditions specified by law and authorized by the UGent Institutional Ethical Committee on Experimental Animals. The animal facility, located at the VIB-UGent Center for Inflammation Research, Ghent, Belgium, operates under the Flemish Government License Number LA1400536.

Note that full information on the approval of the study protocol must also be provided in the manuscript.

Flow Cytometry

Plots

Confirm that:

☒ The axis labels state the marker and fluorochrome used (e.g. CD4-FITC).

☒ The axis scales are clearly visible. Include numbers along axes only for bottom left plot of group (a 'group' is an analysis of identical markers).

☒ All plots are contour plots with outliers or pseudocolor plots.

☒ A numerical value for number of cells or percentage (with statistics) is provided.

Methodology

Sample preparation	1) Spleens were isolated from female C57BL/6 mice and processed through a 40 µm cell strainer. Red blood cells were removed using Red Blood Cell lysis buffer (eBioscience) according to the manufacturer's instructions. A total of 2 x 10^6 splenocytes were transferred in a round bottom 96 well plate (200µl volume) and ex vivo restimulated with 1µg/ml of the lmon_0149 peptides YSYKFIRV (GenScript) and QVFEGLYTL (in house prepared by solid phase synthesis) or the OVA-derived peptide SIINFEKL (Eurogentec, Seraing, Belgium) as control in the presence of a protein transport inhibitor cocktail of Brefeldin A and Monensin (eBioscience). Following 37°C incubation for 5 hours, cells were stained for 30 min with fixable viability dye Aqua (Thermo Scientific, 1:400 #L34965, lot 2298184), together with Fc block (CD16/32) to block nonspecific FcR binding (TruStain FcX Biolegend 1:200 #101320, Lot B330502) in PBS buffer. After washing the cells once with FACS buffer (PBS supplemented with 1% BSA and 0.09% sodium azide), a surface staining was performed with antibody CD8a (53-6.7) APC (Biolegend 1:100) OR with antibody CD8a (53-6.7) FITC (Biolegend 1:100) and antibody TCRbeta (H57-597) APC (Invitrogen 1: 100) in FACS buffer. After washing with FACS buffer, cells were then fixed with BD CytoFix for 20 min at room temperature, and after washing stored in 200µl FACS buffer for 12 hours. After washing with BD Cyotoperm buffer, cells were incubated in Cytoperm buffer including IFN-ɣ (XMG1.2) PE antibody (Biolegend 1:50) for 30 min at room temperature. Finally, cells were washed two times with Cytoperm buffer and kept in 200µl FACS buffer until acquisition.<br><br>2) Human HeLa and HCT-116 cells were first stained with fixable viability dye Zombie green (1:400 #423111, Biolegend, San Diego) and then incubated with Fc block TruStain FcX (CD16/32) to block nonspecific FcR binding (1: 200 #422302, Biolegend). To detect MHC-I and MHC-II, cells were stained with antibodies against HLA-ABC (W6/32) PE (1:100 #311405, Biolegend) and HLA-DR/DP/DQ (Tü39)-APC (1:100 #361713, Biolegend), respectively. Cells were fixed with 4% PFA for 40 min (#15710, Laborimpex).
Instrument	MACSQuant 16 flow cytometer (Miltenyi Biotec, Bergisch Gladbach, Germany).
Software	The acquisition of flow cytometry data was controlled using MACSQuantify 2.13.1 (Miltenyi Biotec). FlowJo version 10.7.1 (BD, Ashland, OR) were used for analyzing flow cytometry data. Three samples that showed a cell viability lower than 50% were excluded from analysis.
Cell population abundance	Antigen-reactive CD8a cells were defined as IFNɣ+ expressing cells after peptide stimulation, and their frequencies were determined among total CD8a cells. Abundances of this population ranged between 0.009% and 4%.
Gating strategy	1) Splenocytes were first gated using FSC-A and SSC-A to exclude debris. Cell aggregates were removed by gating according to FSC-H/FSC-A parameters. Dead cells were excluded from analysis by gating for only live/dead aqua-low cells and then gated for CD8a positive cells. Antigen-reactive CD8a cells were defined as IFN-ɣ+ expressing cells after peptide stimulation, and their frequencies were determined among total CD8a cells.<br><br>2) Cells were first gated using FSC-A and SSC-A to exclude debris. Cell aggregates were removed by gating according to FSC-H/FSC-A and SSC-H/SSC-A parameters. Dead cells were excluded from analysis by gating for only live/dead zombiegreen-low cells and intensity of PE (HLA-ABC) and APC signal (HLA-DR/DP/DQ) were determined.

☒ Tick this box to confirm that a figure exemplifying the gating strategy is provided in the Supplementary Information.
